# Supplementary figures and images for: The impact of trait number and correlation on functional diversity metrics in real-world ecosystems
Source: PLoS One. 2024 Sep 23;19(9):e0306342. doi: 10.1371/journal.pone.0306342 (PMC11419356; doi:10.1371/journal.pone.0306342)

Community

- CDR1
- CDR2
- CDR3
- CDR4
- SEV1
- SEV2

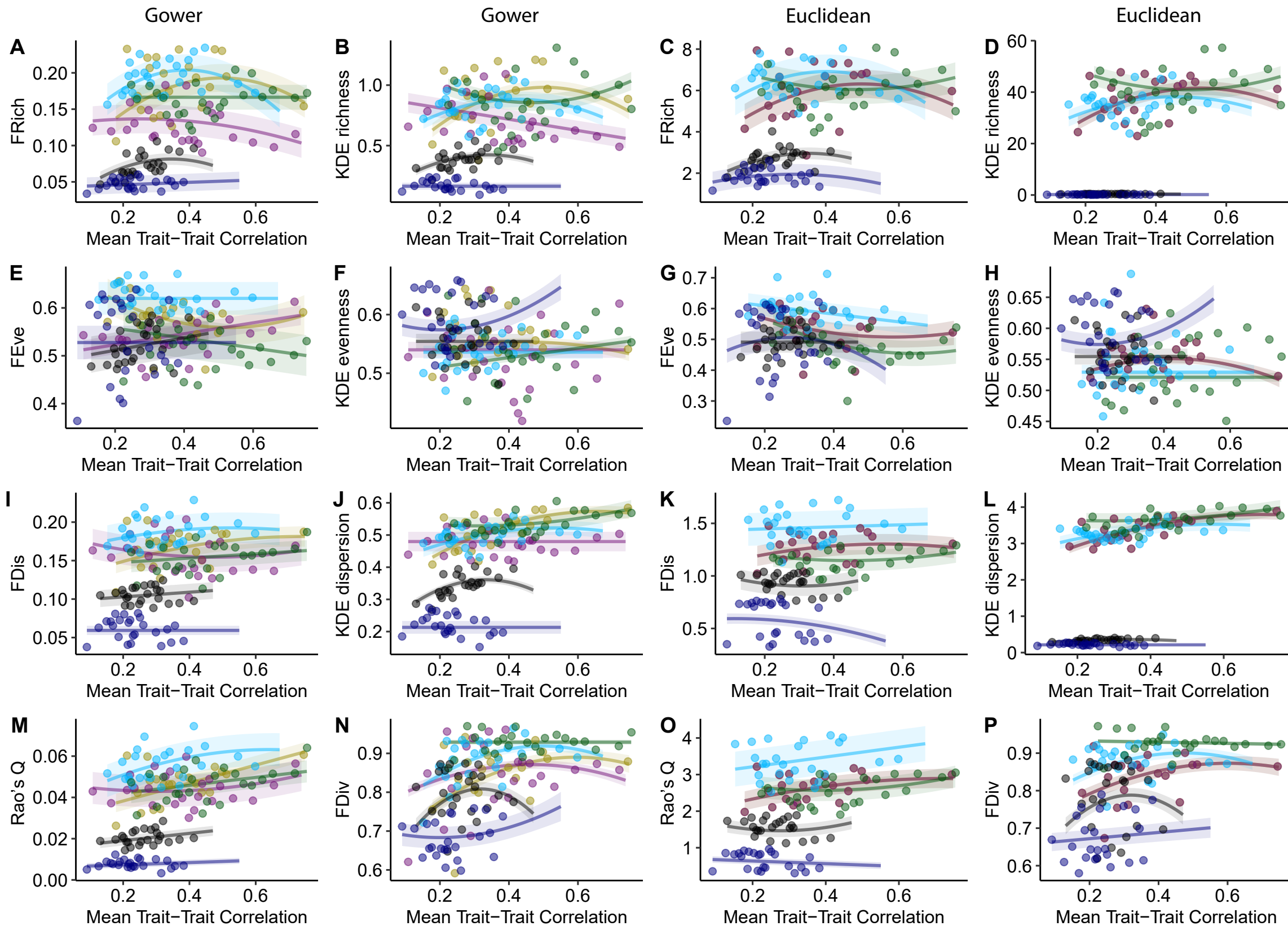

Supplement: S1 Fig — Each point represents the mean value for the given community for a specific number of traits. Solid lines are the predicted fits of the best model and shaded regions are +/- SE of the predicted fit. Different colors represent the six communities used in this study (four experimental communities at Cedar Creek Ecosystem Science Reserve, CDR and two natural communities at Sevilleta National Wildlife Refuge, SEV). N = 1,512 observations for each CDR community; n = 3,402 for SEV1; n = 3,528 for SEV2. (PDF) [file pone.0306342.s001.pdf]

Community

- CDR1
- CDR2
- CDR3
- CDR4
- SEV1
- SEV2

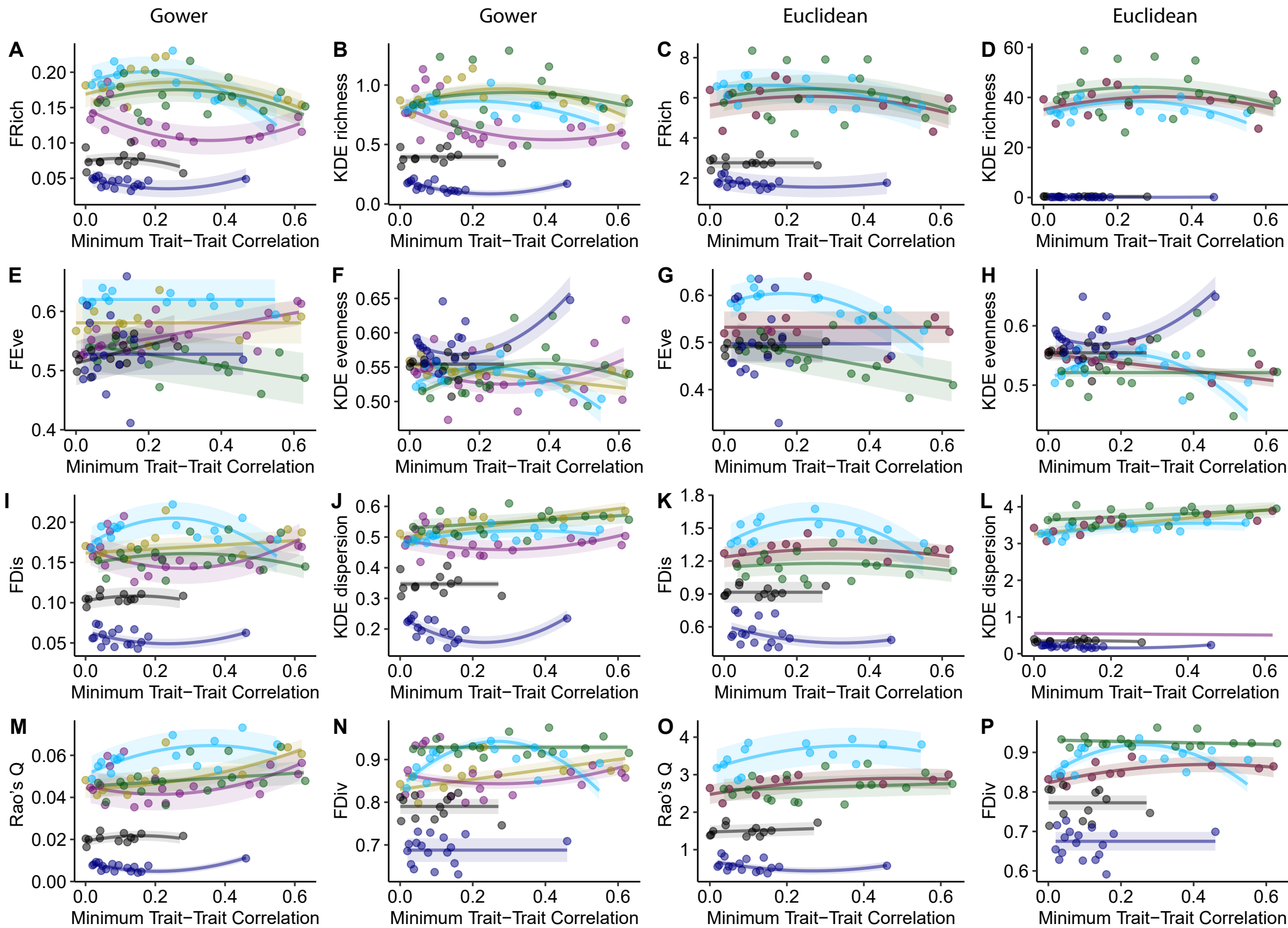

Supplement: S2 Fig — Each point represents the mean value for the given community at that correlation. Solid lines are the predicted fits of the best model and shaded regions are +/- SE of the predicted fit. Different colors represent the six communities used in this study (four experimental communities at Cedar Creek Ecosystem Science Reserve, CDR, and two natural communities at Sevilleta National Wildlife Refuge, SEV). N = 1,512 observations for each CDR community; n = 3,402 for SEV1; n = 3,528 for SEV2. (PDF) [file pone.0306342.s002.pdf]
