# Supplementary material for: The impact of trait number and correlation on functional diversity metrics in real-world ecosystems
Source: PLoS One. 2024 Sep 23;19(9):e0306342. doi: 10.1371/journal.pone.0306342 (PMC11419356; doi:10.1371/journal.pone.0306342)
Supplement: Attachment — Submitted filename: Response to reviewers.docx [file pone.0306342.s003.docx]

*We thank the two reviewers for their comments. Their reviews were most helpful for improving the clarity of the text and focusing the aims of our study. We have responded to each individual comment. In most cases, we agreed with the reviewers comments and either altered text or changed/added analyses as necessary. In a few instances for which additional analyses were proposed, we have provided justification for the methodology proposed in the Registered Report article and performed for this manuscript. For convenience, we have included in these responses quoted text from our most recent submission. Reviewers comments are shown in standard black text while our responses are in blue italicized text.*

Reviewer #1: ## Review PLOSONE: the impact of trait number and correlation on functional diversity metrics in real-world ecosystems

\# General comments
I review the paper entitled "the impact of trait number and correlation on functional diversity metrics in real-world ecosystems" submitted to Plos ONE journal. In this paper, the authors deal with a timely question in functional ecology related to the choice of functional traits to calculate functional diversity indices. Indeed, given the development of trait measurements in ecology, trait-based approaches are more and more common to go behind taxonomic diversity. A key aspect of these approaches is the sensitivity of functional diversity indices to the choice of functional traits (e.g. number and correlation).
In this paper, the authors propose to test the sensitivity of 8 functional diversity indices on real community of plants. Thus, they test the behavior of functional diversity indices to the number of functional traits used to describe the community. In addition they also test how the traits correlation influence the FD indices. They found that some indices decrease when the number of traits increased, while other increase and some are not changing. They also found that the trait-trait correlation have no significant influence of the functional indices.
The more I read the paper the more I had some problems to understand the real aim of this paper. When the authors claim L. 75 "Therefore, functional diversity could differ among replicate plots or sites simply because of the number or types of traits used to calculate the metric without any underlying ecological basis." and L. 407: "Some metrics may be unreliable measures for comparing functional diversity among communities since comparisons are dependent upon the number of traits used to calculate them." OK, I agree, but does people really compared FD of communities calculated on different number of traits ? I would not do it in any case, whatever the metrics give similar or different values. It is just like comparing apple and pear.
As a general comment, I would say that the authors pointed out a real question in their introduction, but the analyses and interpretations are not sufficient to answer the question. The main problem here is the lack of analyses on the type of traits, only the number is taking into consideration. L.104 "The field lacks clear guidelines for researchers to follow when choosing the number and types of traits to include when calculating functional diversity metrics." Although this statement is not completely true, this study do not help to solve it. There is no conclusion on which traits and how many traits is required. So far, this study gives hint about sensitivity of indices on trait number. Moreover, there is no clear explanation about ecological processes behind, since analyses performed here pointed out mathematical properties of the different indices to trait number.

*Thank you for this comment as it has helped us clarify the aims of this study. Though we might have found a conclusive answer as to the specific number of traits required to generate a robust comparison of rankings among communities, our results generated no such obvious conclusion. We did report results and include recommendations for using certain metrics for which rankings among communities were inconsistent across the range of number of traits used to calculate them (such as FEve). We agree that the focus of this paper is primarily to test the mathematical properties of these metrics and we believe that our study represents meaningful progress in the field by, for the first time, using real grassland data in this investigation instead of simulated data.*

About the sensitivity of FD to trait number: This is a very exiting question ! However, I am not sure I found a satisfying answer in this paper, or at least given the analyses the authors performed here. The fact that FD indices varies with the number of traits is not surprising but more importantly, I do not know how to interpret it! The authors seem to interpret it as a weak point of the method since they focus on FDis (1st paragraph of discussion), which do not vary with the number of traits. They justify it by the fact that : "This suggests that FDis provides reliable values across different communities and sets of traits, making it a potentially valuable tool for assessing patterns of functional diversity across communities and ecosystems." This is not true. For me, what is important is the ranking of communities across trait number. The question of community ranking is the key aspect here. Indeed, more than the values itself, most of the study in community ecology are based of the relationship of FD indices between communities. I would like to see this part more developed.
*We agree that the relationships of FD indices among communities is the most important factor to consider. To that end, the first paragraph of the discussion section highlights the suite of metrics that maintain consistent relationships among communities across the range of trait numbers L 435-441: “Similarly, Rao's Q, KDE dispersion, and KDE richness maintained consistent ordered rankings of metrics among communities across the range of trait numbers for both Gower and Euclidean dissimilarity matrices, although differences between communities were magnified at higher numbers of traits when using Euclidean matrices. Other metrics (FEve, KDE evenness, FDiv) had less consistency across the range of traits used to calculate them as relative rankings of different communities changed with the number of traits used in constructing the metrics.“*

*L. 463-469: “Specifically, FRich, FEve, and KDE evenness showed crossing slopes among communities (i.e. ranking of communities changed with the number of traits) for both Gower and Euclidean distance matrices. The inconsistency of communities’ relationships to one another across the range of the number of traits raises concerns. For example, community CDR4 had a greater FEve than community SEV1 when using four traits, but CDR4 had a smaller FEve than SEV1 when calculated with eight traits. Such discrepancies have the potential to introduce discordant results in the literature, even when otherwise identical studies have been conducted.”*

*In addition, our recommendations support the use of metrics that maintain consistent rankings of communities and suggest supplementary analyses for studies using metrics for which rankings among communities are inconsistent L 528-533: “Based on our findings, we recommend use of FDis, KDE dispersion, and/or Rao’s Q in analyses of functional diversity as all of these measures provide consistent results among communities at all numbers of traits tested. Additionally, due to the inconsistency of evenness metrics with respect to community rankings, we strongly recommend that any use of FEve or KDE evenness metrics include supplemental analyses to test whether results are consistent with different numbers of traits used to calculate them.“*

Moreover, FD indices, at least some of them, are highly influenced by the species richness. Most of community ecologist working of FD used null models where they compared the observed values to expected values. This would be more interesting to analysis and interpret, because SES will be comparable between community irrespective of their species richness, but also between number of traits with clear null hypothesis that a useful SES values of a FD index should be stable.
*We recognize that use of richness-based null models is helpful in some contexts in order to isolate the effects of traits on FD separate from effects of species richness. In this study, we specifically chose an approach agnostic to species richness as the focal objective was to resolve the predictability and reliability of metrics across ranges of trait number and correlations as opposed to investigating which metrics were more or less informative than simple species richness. Consider, if a species-based null model was chosen as the best model through model selection in our paper, we could conclude that the FD metric is a worse predictor than species richness but would have learned nothing about reliability of FD metrics. To that end, we do include a null, intercept-only models which test whether or not any change in metrics occurs across the variables which we test (number of traits and correlation). Many papers in the literature take a similar, trait-agnostic approach when questions focus on comparing functional diversity among communities as opposed to comparing relative explanatory power of richness among communities (e.g. Zihao et al. 2021, Mao et al. 2022).*

From a methodological point of view, I am not sure whether the FD indices are calculated directly using traits or after PC(o)A. L. 280, the authors claim that they "used dimensionally reduction where necessary". but later in the paragraph, they said L.291 they "calculated each metric using all possible combination of two traits up to all possible combinations of the maximum number of traits".There is also no information on whether traits are center/scale before calculated FD indices. I got a bit lost here, or try to explain better what you want to do. This might have strong implications, since if not scaled, traits might have different weight.

*We have now added additional text to clarify our methodology. There seems to be confusion about dimensionality reduction and how we looked at the impact of trait number on the metrics. The text now reads:*

*L 281-287: “To understand the impact of trait number on functional diversity, each functional diversity metric was calculated using all possible combinations of two traits up to all possible combinations of the maximum number of traits at each site. For example, at Sevilleta there are 10 different traits so there are 45 2-trait calculations, 120 3-trait calculations, 210 4-trait calculations, and so forth up to 10 9-trait calculations and 1 10-trait calculation. This allows us to focus on the impact of trait number independent of the constituent set of traits used to calculate the metric.”*

*L288-297: “To calculate the five metrics using the FD package, we first calculated a species-trait distance matrix using both Gower (categorical and continuous traits) and Euclidean (continuous traits only) distances. These distance matrices were calculated with both scaled and centered and non-scaled trait data for each community. Centering was done by subtracting the trait mean from each observation and scaling was done by dividing the centered traits by their standard deviations (as in the FD package). These distance matrices along with a species-abundance matrix are the input for the FD package. The FD package performs a principal components analysis on the full species-trait distance matrix. Dimensionality reduction only occurs for FRic and FDiv metric calculation. For all FRich and FDiv analyses, we hold the number of dimensions equal to 2, similar to Legras et al. [20].”*

*We have added a table to the supplemental material which shows the model selection results when unscaled data are used (i.e. Table 3 in the main text but using unscaled data). The best fit functional forms changed for just a few of the metrics and overall the changes do not affect our interpretation of the results.*

If the authors used traits to calculate indices; I disagree with their discussion. L. 407, they said that some FD indices are not suitable because the ranking changes given the number of traits. and later, L.415: "This is particularly concerning given the often arbitrary nature of selecting the number of traits used in a study.". This is not surprising but instead, it can be useful, meaning that some traits (or combination) add some information. Otherwise why not using only body size... Moreover there is no explanation why such differences happened. Does some specific type of traits bring new information and change the patterns ? Here, and more generally in the discussion, there is a lack of ecological explanations of the results. A great advantage of working on real community would be to correlate outputs of indices (mathematics) to ecological process or at least to the different type of traits/species.
*Thank you for this comment. We agree that the primary results from this work represents mathematical properties of these indices and therefore we do not try to make unsupported ecological conclusions. In our analyses, every combination of traits exists at each of the values of number of traits on the x-axis. Therefore, since every trait is equally represented across the range of x-axis values, the fact that the values of metrics change across the range of values is not due to ecological processes of different traits being used to calculate the metrics. In other words, the trait-number analyses do not assess relative contribution of certain traits because it is agnostic of trait identity. Conveniently, the trait correlation analyses provide information as to how the types of traits used impact the metrics, albeit with a focus on trait correlation as opposed to trait identity per se. Though we can speculate as to how certain traits drive variability in certain metrics (e.g. line 446-450 which reads “These differences in findings might be attributed to the inherent complexity and noise present in real-world data. Real communities often contain anomalous species with outlier trait values (e.g. a gymnosperm among angiosperms, a tree seedling among herbaceous plants, or other rare species outlier values), which can exert considerable influence on evenness indices.“), conclusions about how these metrics relate to ecological properties or specific conclusions about certain traits or species are outside of the scope of this work and would require a wholly different study design. However, we agree on the importance of such work.*

In addition, when we have several traits, a common strategy is to make a PCA/PCoA, then it would have been wise to test if the number of traits changes the results, not on the raw data but on the FD indices calculated after using a PCoA. FD indices would be calculated on a PCoA with same number of dimension. Such approach would be more relevant.

*Though use of PCA/PCoA for dimensionality reduction is common in studies using functional diversity metrics, it is also common to not use dimensionality reduction (e.g. Thakur & Chawla 2019, Zuo et al. 2021, Biswas et al. 2019, Niu et al. 2015). In particular, KDE metrics do not use dimensionality reduction in their calculation and in order to make metrics most comparable to each other, we also did not use dimensionality reduction in the FD metrics except for those metrics that require it. Moreover, Legras et al. 2020 showed in their supplemental material that metrics are very similar regardless of the extent of dimensionality reduction.*

About the trait-trait analysis, the effect of traits correlation was calculated only with 4 traits (L.295), but I am a bit surprising of this choice. Please justify it. All this lack of details (including my previous remarks) make that the trait-trait analyses have weak support so far.

*Four traits was chosen as a compromise between the minimum number of traits likely to be used to calculate these indices and an effort to reduce noise in the evaluation of minimum and maximum trait correlation. Max and min include all possible combinations of four traits and therefore all possible pairwise combinations of two traits to generate max and min. Including greater numbers of traits in these analyses would only add additional noise to potential signals since every trait beyond the two minimum or maximum correlated are nothing but additional noise (though theoretically could change mean correlation in modest ways). We have added text to justify L. 307-310: “Only combinations of four traits were used as a balance between reduction of noise in the evaluation of minimum and maximum correlation (calculated as the pairwise correlation of just two traits) and the lower end of the number of traits likely to be used to calculate these metrics in the literature.“*

Finally, there is no mention of the trait selection. The choice of the traits and its selection seems to be randomized (e.g., L; 294: "10 different traits so there are 45 2-trait calculations, 120 3-trait calculations[...]). Do it mean that only the number but not the identity matter? And should we expect similar results if the type of traits is different ? For instance the authors L. 402 explain differences with other studies by the number of traits, but in any case we have information of the type of traits used.
*Our trait correlation analyses are agnostic of trait identity per se, but assess the question of the type of trait as quantified by the correlation or traits. In this way, we are able to isolate the properties of input data most likely to be mathematically relevant to the calculation of these indices. Literature using these metrics often includes the traits that we used in our study, but also includes additional traits not used in this study. Therefore, our method of testing the correlation of traits to each other agnostic of trait identity has the greatest opportunity to inform future research which may or may not be using the specific traits we have available.*

\# Minor comments
L. 46. Do you have example of such studies ? This is quite dangerous approach

*We intended for this statement to imply qualitative comparisons among studies but we agree that as written it implies quantitative comparison. We have changed this sentence to more specifically reflect our meaning. L 46-50: “We recommend that future use of evenness metrics include sensitivity analyses to ensure results are robust to the number of traits used to calculate them. In addition, we recommend use of FDis, KDE dispersion, and Rao’s Q when ecologically applicable due to their ability to produce consistent rankings among communities across a range of the numbers of traits used to calculate them. “*

L. 75. reference is needed

*This reference has been added*

L. 75. "Therefore, functional diversity could differ among replicate plots or sites simply because of the number or types of traits used to calculate the metric without any underlying ecological basis." But if the trait differs, its means that the ecological processes also differ. here the problem is not about metrics, but more about what we mean by "functional diversity". In another way, it is NOT because we got similar result with different set of traits, that the metric is better (or true).

*Since all of the combinations of traits are used at each of the levels of number of traits used to calculate them (i.e. the x-axis of figure 1), our results show exactly that the rankings of communities to each other can change even with the same sets of traits used to calculate them. This is not an ecological property but a property of the metrics.*

L. 81. "it is important to determine the conditions under which they reflect ecological processes as opposed to mathematical patterns." I agree, but this aspect is not solve in this paper. for that a analysis on the trait type and relevance with ecological processes is needed not on the metrics. Metrics is just a way to calculate but it will in any case represent ecological processes.

*We agree that our paper does not answer the question of which traits reflect certain ecological processes. Instead, our goal is to determine what hidden mathematical patterns might obscure the results of other studies that specifically address questions of ecological processes. To that end, the sentence referenced above reflects the purpose of this paper.*

L. 80. "As use of multi-trait functional diversity increases, it is important to determine the conditions under which they reflect ecological processes as opposed to mathematical patterns." OK, but this is not what the authors are doing here.

*Our response is addressed in the above comment. We are indeed determining the conditions under which metrics reflect mathematical patterns so that future studies may focus on ecological processes.*

L. 122. replace value by metrics

*We have made this change.*

L. 137: To refine objective of the paper I suggest to clarify the objective 1 (L. 137) the term "vary" need to be better explained.

*We have modified this to ask whether functional diversity indices exhibit predictable patterns across the range of trait numbers and correlation. We believe this better reflects the aims of our study. L. 141-142: “(1) Do functional diversity metrics exhibit specific patterns with respect to the number and correlation of traits used? “*

Moreover, I do not understand well this objective since the expectations are mathematical properties of the indices. Maybe can be wise to adapt them to the specific trait/species of the grassland?

*Indeed, the expectations are mathematical properties of the indices. It is important to understand these first before answering ecological questions regarding specific traits and species which are often addressed in ecological papers with functional diversity metrics.*

L. 228/ Are the traits scaled/centered?

*All traits are scaled and centered to z-score for results in the main text. We also reproduced Table 3 using unscaled traits which is in the supplemental material as Table S1. We have added text to this point in our methods. In our revision process, we discovered an issue in our code involving package defaults which caused portions of our metrics to be calculated without scaling. This issue has been fixed with minimal change to results (see Table 3).*

L. 280/ What means "where necessary"?

*We have replaced this line with an extended explanation of methods calculation including that FRic and FDiv require dimensionality reduction for there their calculation. These are the two metrics for which dimensionality reduction was necessary.*

L. 318: "Adequate" avoid such vague vocabulary.

*The following sentences in this paragraph further clarify our meaning of adequate. Therefore, we have removed this sentence for clarity.*

L. 328: How many communities have only one species ?

*We have clarified that two plots across all communities have only one species. L299-302*

*“Further, when running the analyses for FRic, FEve, and FDiv, one plot from each Sevilleta communities with only one species present were removed because FRic, FEve, and FDiv are undefined in monoculture communities.“*

L. 331: this sentence is not clear

*We agree and have deleted this sentence*

L. 332: "some": which?

*We have changed this sentence to highlight the FDis metric.*

L. 336: "relationships of communities to each other" rephrase it

*Rephrased for clarity.*

L. 332: The reference to null model is not clear here. Do you mean that the relationship is not significantly different that expected under null model?

*Rephrased for clarity.*

L. 362: I did not understand the goal of this analysis here. it has not been mention in introduction, and barely in method.

*Both distance matrices are assessed in this study in order to provide comprehensive information to the scientific community. Both distance matrices are used extensively in the literature and this paper would be incomplete if we addressed only one distance matrix and not the other.*

L. 376. The authors focused on 1 index, but it is not explain why here.

*The remainder of the paragraph addresses the other seven indices. Just two sentences in this paragraph are focused on FDis but we discuss this metric first because it was the only metric with “no correlation with the number of traits used”(L.432).*

L. 402. Here a discussion about the type of trait is also needed.

*As noted in an above comment, each trait is represented equally at each level of the number of traits used to calculate the metrics. Therefore, this analysis is agnostic to the type of traits used and is not informative to that point.*

L. 407. I do not agree. Here some analyses of SES are required to conclude

*We are unclear what the acronym SES refers to. Given the context, we believe that we have adequately shown that for some metrics comparisons among communities are dependent upon the number of traits used to calculate them.*

L. 412. Can you provide some explanation why such discrepancies of FEve? Does it come from mathematical issues or ecological process?

*We are focusing on mathematical issues given our trait-agnostic approach. We discuss potential reasons why outlier values might have increased influence on evenness indices in previous L. 446-450: “These differences in findings might be attributed to the inherent complexity and noise present in real-world data. Real communities often contain anomalous species with outlier trait values (e.g. a gymnosperm among angiosperms, a tree seedling among herbaceous plants, or other rare species outlier values), which can exert considerable influence on evenness indices.“*

L. 450: I do not agree about this distinction between continuous/categorical data. Continuous are more common and available on a larger range of species. Moreover filling gap procedure are also well-know and work quite well ! Instead I would expect a real discussion on why indices are working on both type of distance.

*It is unclear what is meant by a real discussion on why indices work well on both distance matrices. We have a small section of the discussion dedicated to this point and the clear conclusion is that they both work similarly and it is unclear how we could determine why they both work similarly. Additionally, continuous traits are notoriously difficult to gap fill even with modern methodology (e.g. Poyatos et al. 2018, Joswig et al. 2023). Putting aside the propensity for imputation methods to generate nonsensical values, imputed continuous trait values are just educated guesses impossible to verify without on-the-ground measurement. Categorical traits, on the other hand, can be easily collected by novices and verified by experts multiple time over (e.g. multiple researchers searching the internet to determine whether a species is a forb or graminoid). We have changed L 508 from ‘easier to gap fill’ to ‘more reliably gap-filled’ since the easiness of a task is subjective.*

Fig: in some cases, not all communities are visible, maybe you can use thinner lines and/or transparency

*We have changed the aesthetics of the figures including thinner lines, smaller points, and more transparency of both lines and points.*

Please check your references, some are in different style.

*Thank you, we have corrected these.*

Reviewer #2: I reviewed the manuscript ‘The impact of trait number and correlation on functional diversity metrics in real-world ecosystems’ submitted for publication in PLOS One. The authors use vegetation data from natural and experimental communities to explore how trait number and correlation impact on various functional diversity metrics. The Introduction is extremely well written and clearly explains the importance of this study. The Discussion is also great and provides useful guidelines for researchers analysing functional diversity. Since the paper is rather methodological I found that the Methods and Results part could benefit with some further explanations and clarifications to further enhance the clarity and readability of the study. Please see my suggestions below:

Methods:
Line 161 – it’s a little difficult to follow how many sites and grasslands there were (e.g. in line 163 could be understood that each grassland had a site with natural and a site with planted community), maybe start the site description with the fact that there were two sampling sites and then mention how many communities and whether natural or planted were sampled in each site

*We agree and have revised this text for clarity.*

Lines 165, 173 – add approximate coordinates for the sites

*We have added this*

Line 182 – add which year

*We have added this*

Line 185 –unclear if the 48 plots were in total for four communities, or were there 48x4 plots? explain somewhere how the two communities were located and how they differed from each other

*We have clarified this. L 187-189: “At Cedar Creek, we used community composition data from all 16-species plots in a biodiversity, CO_2_, and nitrogen addition experiment (BioCON, n = 48; 12 plots for each CO2-N combination).“*

Line 205 – the text in line 202 gives impression that data was collected only in 2018, so it reads a little strange here that the 2018 was selected

*Yes, this was confusing as written. We removed the reference to the trait data collection timing in the community composition data section to try to alleviate this confusion.*

Line 249 – remove one ‘then’ from the sentence

*Done.*

Line 258 – missing part of the formula

*This is the formula as it appears in Villeger 2008*

Line 266 – this is also mentioned in lines 276-277, maybe not needed here

*Agreed. This has been removed.*

Line 305 – unclear here how the models were run for each community if the min/max/mean trait correlations were also calculated for each community (so there’s a single value of explanatory variable)

*We have added text clarifying the methods for min, max, and mean trait correlation.*

Results
Lines 317-329 – this would fit better in the methods, some of this information is already mentioned earlier in the text

*Due to the registered report format, we think this section makes most sense at the beginning of the Results section. If the editor disagrees, we are fine with moving it to the Methods section.*

Line 332 – if i understand correctly then only null model shows no relationship? if that’s the case then use ‘i.e.’ instead of ‘e.g.’

*We agree and have rephrased this sentence for clarity.*

Line 335 – are these results shown somewhere? add reference to figure

*We have added reference to the appropriate figure.*

Line 336 – the part ‘relationships of communities to each other’ is unclear, maybe just use the phrasing that is currently in the parentheses (and maybe use ‘from low to high functional diversity metric values’ for clarity)

*We agree and have changed this sentence accordingly.*

Line 344 – is this correct ‘depending upon the number of traits used to calculate them’? isn’t this the relationship that is being tested? maybe rephrase to clarify, consider also adding an explanation to the Methods what rankings were compared
*This sentence was confusing and redundant as written. We have simplified for clarity.*

Table 3
Unclear what is ‘functional form’.

*We have deleted that clause to mitigate confusion.*

Add some further explanations in the table header as to how to read the table, it’s not very intuitive that the models that are compared are per each metric, distance matrix and predictor combination (within a row, but considering the same distance matrix, right?), and not clear that the 6 (for each distance measure) models are for different communities.
Explain also what mean, maximum and minimum correlations mean, so the table would be understandable without necessarily reading the text.

*We have added additional text describing this table.*

Line 352 – add a reference to a table or figure

*Reference to figures has been added.*

LIne 354 – use ‘an Euclidean’

*We have made this change.*

Line 360 – unclear what is the percentage from, models?

*We have added test for clarity.*

Discussion:
Line 381 – the part ‘though their magnitude was affected by input trait number’ could be removed since this is said also at the end of the sentence and currently makes the sentence difficult to read

*We have made this change.*

Line 412 – use ‘community CDR4’ to remind the reader again what these acronyms stand for

*Agreed. We have made this change.*

Line 428 – what is ‘and unresponsive relationship’, do you mean ‘or no relationships’

*We have changed this to ‘null relationships’ to match nomenclature in the rest of the MS.*

Line 444 – could this be better as ‘Gower or Euclidean dissimilarity’?

*We agree and have made this change.*

Fig. 1
Are some of the communities not shown, e.g. in J, or are they covered up by each other? Maybe with a bit thinner lines and smaller points they could be visible?
*We have changed the aesthetics of the figures including thinner lines, smaller points, and more transparency of both lines and points.*

Supplementary materials
The data collection protocols include Konza, but since this data was not used in the current study, maybe not needed to include here to avoid confusion

*We have made this change.*

*References cited in reviews*

*Biswas SR, Mallik AU, Braithwaite NT, Biswas PL. Effects of disturbance type and microhabitat on species and functional diversity relationship in stream-bank plant communities. Forest ecology and management. 2019 Jan 15;432:812-22.*

*Joswig JS, Kattge J, Kraemer G, Mahecha MD, Rüger N, Schaepman ME, Schrodt F, Schuman MC. Imputing missing data in plant traits: A guide to improve gap‐filling. Global Ecology and Biogeography. 2023 Aug;32(8):1395-408.*

*Mao W, Sun Z, Forrestel EJ, Griffin‐Nolan R, Chen A, Smith MD. Using local and regional trait hypervolumes to study the effects of environmental factors on community assembly. Ecosphere. 2022 Oct;13(10):e4253.*

*Niu K, He JS, Zhang S, Lechowicz MJ. Grazing increases functional richness but not functional divergence in Tibetan alpine meadow plant communities. Biodiversity and Conservation. 2016 Nov;25:2441-52.*

*Poyatos R, Sus O, Badiella L, Mencuccini M, Martínez-Vilalta J. Gap-filling a spatially explicit plant trait database: comparing imputation methods and different levels of environmental information. Biogeosciences. 2018 May 4;15(9):2601-17.*

*Thakur D, Chawla A. Functional diversity along elevational gradients in the high altitude vegetation of the western Himalaya. Biodiversity and Conservation. 2019 Jul 30;28:1977-96.*

*Zihao Z, Jihua H, Nianpeng H. Predictability of functional diversity depends on the number of traits. Journal of Resources and Ecology. 2021 May;12(3):332-45.*

*Zuo X, Zhao S, Cheng H, Hu Y, Wang S, Yue P, Liu R, Knapp AK, Smith MD, Yu Q, Koerner SE. Functional diversity response to geographic and experimental precipitation gradients varies with plant community type. Functional Ecology. 2021 Sep;35(9):2119-32.*
