# Supplementary material for: The impact of trait number and correlation on functional diversity metrics in real-world ecosystems
Source: PLoS One. 2024 Sep 23;19(9):e0306342. doi: 10.1371/journal.pone.0306342 (PMC11419356; doi:10.1371/journal.pone.0306342)
Supplement: Attachment — Submitted filename: Reviewer comments Round 2.docx [file pone.0306342.s004.docx]

*Responses to Reviewer’s comments are below in blue italicized text.*

Reviewers' comments:

Reviewer #2:

The authors have done a great job in revising the paper and addressing reviewers' comments. I only have a few minor suggestions to improve the clarity of the text in a few places:

Line 50 – the acronyms are not defined in the abstract, better to write out or define in lines 44-45

*We agree and have made this change.*

*“We found that most metrics were sensitive to the number of traits used to calculate them, but functional dispersion (FDis), kernel density estimation dispersion (KDE dispersion), and Rao’s quadratic entropy (Rao’s Q) maintained consistent rankings of communities across the range of trait numbers” lines 43-46*

Table 3
Line 375 – in the table header the example for Frich, Trait number and Gower (first line in the table) doesn’t fit with the results in the table (text says 2 for linear and 4 for quadratic, but table has 3 and 3 – i think the text hasn’t been updated for the new version of the table)
In the table use ‘FRich’, ‘FDis’, ‘FEve’ and ‘FDiv’ like in the rest of the manuscript
KDE diversity measures are not capitalised in the the text (e.g. ‘evenness’), but are here and on the figures – better to use the same spelling everywhere

*We agree and have made these consistent.*

Line 385 – this is the first time CDR is used, but it’s not defined anywhere

*We have now defined CDR and SEV in lines 170 and 178, respectively.*

Fig. 1 and Fig. 2 have different legends, but I guess these should be the same?

*These are now consistent.*

Fig. 2, Fig S1, Fig S2
Lines 399, 407, 415 – CDR and SEV are not defined

*We have now defined these in lines 170 and 178.*

Line 527 – perhaps this section could be combined with the previous one (e.g. using heading ‘The ecological significance of methodology and recommendations’ or something similar) since some recommendations are already given in lines 519-526

*We like this idea and have edited it accordingly. Line 515*
